# The Hormetic Adaptative Capacity and Resilience to Oxidative Stress Is Strengthened by Exposome Enrichment with Air Cold Atmospheric Plasma: A Metabolome Targeted Follow-Up Approach

**DOI:** 10.3390/biomedicines13040949

**Published:** 2025-04-12

**Authors:** Lucía González-Llorente, Miguel Andrés-Gasco, Macarena Alba Gil Aranda, Rubén Rabadán-Ros, Rubén Zapata-Pérez, Estrella Núñez-Delicado, Nerea Menéndez-Coto, Claudia García-González, Francisco Javier Baena-Huerta, Ana Coto-Montes, Enrique Caso-Peláez

**Affiliations:** 1 UCAM HiTech Sport & Health Innovation Hub, Universidad Católica de Murcia, Guadalupe de Maciascoque, 30107 Murcia, Spain; 2 System and Precision Medicine Unit, Hospital Ribera Covadonga, 33204 Gijón, Asturias, Spain; 3 Health Sciences PhD Program, Universidad Católica de Murcia UCAM, Campus de los Jerónimos nº135, Guadalupe de Maciascoque, 30107 Murcia, Spain; 4 Research Group of Metabolism and Gene Regulation, UCAM HiTech Sport & Health Innovation Hub, Universidad Católica de Murcia, Guadalupe de Maciascoque, 30107 Murcia, Spain; 5 Research Group of Molecular Recognition and Encapsulation (REM), Health Sciences Department, Universidad Católica de Murcia (UCAM), Campus de los Jerónimos 135, 30107 Guadalupe, Spain; 6 Department of Morphology and Cell Biology, University of Oviedo, 33006 Oviedo, Asturias, Spain; 7 Research Group Oxidative Stress Knowledge and Advanced Research (OSKAR), Instituto de Investigación Sanitaria del Principado de Asturias (ISPA), 33011 Oviedo, Asturias, Spain; 8 Instituto de Neurociencias del Principado de Asturias (INEUROPA), 33006 Oviedo, Asturias, Spain; 9 Instituto de Investigación Sanitaria del Principado de Asturias (ISPA), 33011 Oviedo, Asturias, Spain

**Keywords:** negative air ions (NAIs), oxidative stress, metabolomics, cold atmospheric plasma (CAP), exposome, antioxidant capacity, hormesis, cellular resilience

## Abstract

**Background/Objectives**: The exposome, encompassing all environmental influences on health, plays a pivotal role in oxidative stress-related diseases. Negative air ions (NAIs), generated via cold atmospheric plasma (CAP), have been proposed as potential modulators of oxidative resilience. This study aims to investigate the metabolic adaptations induced by prolonged exposure to an NAI-enriched environment in mice, focusing on its effects in oxidative stress markers and energy metabolism in liver and blood. **Methods**: Twenty male C57BL/6J mice were divided into four groups: two experimental groups exposed to NAI-enriched air generated by an Air Cold Atmospheric Plasma–Nanoparticle Removal (aCAP-NR) device for either 18 days (short-term, ST) or 28 days (long-term, LT), and two control groups without exposure. Targeted metabolomics was performed in whole blood and liver using ultra-high-performance liquid chromatography–mass spectrometry (UHPLC-MS). Statistical and pathway analyses were conducted to assess metabolic alterations. **Results**: Metabolic profiling revealed significant shifts in oxidative stress-related pathways, including enhanced glutathione metabolism, reduced lipid peroxidation, and modulation of purine metabolism. Short-term exposure led to increased mitochondrial efficiency and energy homeostasis, while long-term exposure induced adaptive metabolic reprogramming, with higher inosine levels suggesting enhanced antioxidant and anti-inflammatory responses. No adverse effects on systemic or hepatic health markers were observed. **Conclusions**: NAI exposure via aCAP-NR elicits a hormetic response, enhancing metabolic efficiency and resilience to oxidative stress. These findings suggest that controlled environmental enrichment with NAIs may serve as a novel non-invasive strategy for mitigating oxidative damage and improving metabolic health, as hormetic adaptative capacity and resilience to oxidative stress, warranting further translational research.

## 1. Introduction

According to the World Health Organization (WHO), healthier environments could prevent almost one quarter of the global burden of disease [1]. To address this issue, the WHO Health and Environment Program fosters cross-sector collaboration to improve health by tackling environmental determinants of disease, such as pollution and climate change. This program aligns with the goals of the Horizon Europe program and the Human Exposome Project, which also prioritize health and environmental sustainability, analysing the intricate interplay between diseases and environmental factors [2,3]. The human exposome encompasses all environmental exposures that affect health throughout life, from conception to old age. It includes all external factors like air pollution, diet, chemicals, and lifestyle choices, as well as internal processes like metabolism and inflammation. Particularly, exposure to nanoparticles and air pollutants has been linked to a range of respiratory and cardiovascular illnesses due to their ability to penetrate deep into the lungs and bloodstream, highlighting the serious health risks posed by air pollution at a molecular level [4].

A significant component of the human exposome is constituted by negative air ions (NAIs) [5], which are electrically charged atoms that naturally exist in the atmosphere, typically through processes like the movement of water or air [6]. NAIs are believed to have potential health benefits, such as a reduction in stress levels, positively affecting respiratory function and the overall well-being through their interactions in different pathways, including amino acid metabolism, anti-inflammatory responses, and antioxidant mechanisms, as well as enhancing energy production [6,7]. However, the evidence on the potential health benefits that NAIs may have been scarce and more research is needed.

Environmental stressors—such as ultraviolet radiation, ionizing radiation, pollutants, and heavy metals—along with xenobiotics, including antineoplastic agents, substantially promote oxidative stress. This occurs due to an imbalance between the production of reactive oxygen species (ROS) within cells and tissues, and the biological system’s ability to detoxify or neutralize these reactive products. While ROS play essential roles in physiological processes, including cellular signalling, they are mainly byproducts of oxygen metabolism. When environmental stressors enhance ROS production without a corresponding neutralization by antioxidants, it leads to oxidative stress, causing cellular and tissue damage [8]. Among the detrimental effects attributed to ROS are lipid peroxidation, which is implicated in pathological processes such as atherosclerosis, asthma, Parkinson’s disease, renal injury, and preeclampsia, among others [9,10,11,12,13].

On the other hand, exposure to certain environmental stressors is not always toxic. In this sense, cells possess the ability to adapt to stressful environments as part of their evolutionary development, showing beneficial effects in response to a low dose of a harmful or stressful agent. This adaptation to oxidative stress, commonly referred to as hormesis, constitutes a vital mechanism through which cells and organisms respond to and manage environmental and physiological changes related to oxidative stress levels.

Cold atmospheric plasma (CAP) has emerged as an innovative technology in biomedicine due to its ability to generate reactive oxygen and nitrogen species (RONS) without causing damage to living tissues, which appear to elicit immune-like responses [14,15]. CAP is a distinct form of ionized gas that is generated through the decomposition of polyatomic gas molecules or the removal of electrons from monoatomic gases. Characterized by its low temperature and non-equilibrium state, CAP adheres to criteria such as quasineutrality, Debye shielding, and plasma frequency, which define its physical properties [14,15,16]. CAP has demonstrated potential across various medical fields, with therapeutic effects including antimicrobial properties, enhancement of wound healing processes, promotion of stem cell proliferation, and improvement of osseointegration, without inducing cytotoxic effects [14,16,17,18]. Due to its versatility and minimal cytotoxicity, CAP is positioned at the forefront of preventive medicine [19], although its physiological effects, particularly in long-term applications, require further research.

Recently, it has been demonstrated that the nanoparticle-free environment in the aerodynamic size range of 0.3 to 10 μm (PM 0.3–10) significantly enhanced protein synthesis and mitochondrial efficiency in peripheral blood mononuclear cells (PBMCs), resulting in increased ATP production and a substantial reduction in protein damage and oxidative stress levels [4]. Additionally, the combination of a nanoparticle-free environment with an enrichment in NAIs by cold atmospheric plasma has been shown to enhance energy capacity, reduce endoplasmic reticulum stress, and activate cellular autophagic clearance in the brain, leading to a marked reduction in key markers of neurodegeneration [20]. NAIs can be achieved using cold atmospheric plasma (CAP), which produces free electrons capable of attaching to gas molecules such as O_2_ and CO_2_, forming ions like O_2_^−^ and CO_3_^−^ [21,22]. The efficiency of this process depends on factors such as gas composition, electric field strength, and discharge characteristics [20,23,24] (REF. A, C, D). In our aCAP-NR system, ionized air is produced in a closed plasma chamber and delivered into the exposure environment without direct plasma contact, ensuring that NAIs are the predominant active component. In developing indirect CAP medical applications, significant progress has been made in understanding the transport of reactive species from CAP sources to different bioavailable media such as water [25], hydrogels, biopolymers [26], liquid aerosols [27], or even only using air [25,28]. Although by indirect applications of CAP all physicochemical characteristics (direct treatment, exposure to high electric fields, ultraviolet radiation) are lost, there are a limited number of RONS that remain, namely the long-lived RONS (H_2_O_2_, NO_2_^−^, NO_3_^−^, and ONOO^−^) [25,29,30,31]. The mechanisms of the biological effects of plasma include changes in the extracellular and intracellular liquid medium, where RONS transferred to the medium play a dominant role in cellular redox signalling through transient or constantly increased concentrations of these reactive species in the cytosol [32,33]. The application of low-intensity plasma stimulates cellular redox signalling, resulting in increased antioxidant capacity, as well as the initiation of repair processes if necessary. The kinetics of biological effects is called hormesis, and in the field of redox biology, it is a general principle of action of reactive species [34,35]. In general, hormetic effects are characterized by stimulation at low doses and inhibition at high doses [36]. In this context, the main objective of the present study is to elucidate the effects of the external exposome enrichment with NAIs on the endogenous adaptative responses by monitoring the metabolome targeted to oxidative damage in mice.

## 2. Materials and Methods

### 2.1. Animal Experiments

Twenty 19-week-old C57BL/6J, mature adult, wild-type male mice were used. Animals were purchased from Charles River (Charles River Laboratories, SA, Barcelona, Spain) and divided into four groups, with each group consisting of five mice: two experimental groups exposed to air enriched with NAIs generated by a device combining indirect air CAP generation and nanoparticle-removal systems (Biow^®^, Biow Exposomics S.L.U., Asturias, Spain), for 24 h a day as a main source of breathing air, as previously described [20], and two control groups which were not exposed to NAI enrichment. Exposed mice were subjected to 18 (short-term, TS) or 28 (long-term, TL) days of exposure, with free access to food and water. After this period, animals were sacrificed by exsanguination via the retro-orbital venous sinus under 4% isoflurane anaesthesia, and anticoagulated blood and liver tissue were collected and snap frozen for metabolomics analyses. All animal experiments were carried out under license A13220911, approved by the Animal Care and User Committee of the University of Murcia.

Both the experimental and control mice groups shared all the room conditions except the atmospheric anionic dilutions. Indeed, long-lived RONS generated by indirect CAP were only applied to the atmosphere in the cage of the experimental group mice. Animals were kept in an air-filtered room at a constant temperature of 24 °C and a relative humidity of 40% under a controlled photoperiod of 12 h of light and 12 h of darkness. The combined aCAP-RN system, acting as an external exposome modifier, was attached only to the cage of the experimental group mice as the main source of breathable air 24/7 with pulsed anionic emissions.

### 2.2. Air Cold Atmospheric Plasma–Nanoparticle Removal (aCAP-NR) Device

NAI exposome enrichment was carried out by combining Air Cold Atmospheric Plasma and a broad-spectrum nanoparticle removal system in a unit developed by Biow^®^ (Biow Exposomics S.L.U., Asturias, Spain), designed and certified in accordance with normatives EN 60601-1-2:2015 [37] and EN 60601-1:2006+AC:2010+A1:2013 [38], as well as complying with directives 2014/53/EU, 2011/65/EU (ROHS) [39], 1907/2006/ED (REACH), and 2012/19/EU [39], ensuring conformity with European safety and electromagnetic compatibility standards. The system integrates an air purification module with a cold atmospheric plasma (CAP) generator, forming a single unit capable of producing controlled concentrations of reactive oxygen and nitrogen species (RONS). These molecules, produced at biologically active yet low concentrations, are transported in a nanoparticle-free laminar airflow [5,6,7,8,9,10,11,12,13,14,15,16,17,18,19,20], facilitating their absorption through the skin and the mucosal surfaces of the respiratory and gastrointestinal tracts [40,41], ultimately entering the bloodstream. The effectiveness of the system relies on the absence of turbulence in the airflow, ensuring the stability and bioavailability of negatively charged ions.

To confirm the production of RONS, spectroscopic measurements were conducted in the ultraviolet–visible (UV-VIS, 200–800 nm) range using a fibre-optic spectrometer (B&W TEK Inc. Models Exemplar-LS, Newark, DE, USA). It was observed that emission within the visible spectrum (400–800 nm) was significantly lower than that in the ultraviolet range (200–400 nm), suggesting a high RONS yield, consistent with previous reports on this system [20]. The aCAP-NR system is based on a dielectric barrier discharge (DBD) configuration operating at atmospheric pressure. Negative air ions (e.g., O_2_^−^, CO_3_^−^) are formed through dissociative electron attachment to gas molecules within a sealed chamber, without direct plasma exposure to the environment. This configuration limits the presence of confounding factors such as heat, ozone, and UV radiation, ensuring that NAIs are the main active agents delivered to the exposure chamber [21,22,23,24].

The source of gas used for indirect CAP generation was atmospheric air, free of nanoparticles as previously filtered by the combined NR system from indoor room air. The electrical supply circuit was based on a multiple-needle electrode configuration within a chamber with holes for ions to exit. The atmospheric air gas, free of nanoparticles, passes through the electric field generated between an upper plate and the inside needles in a fixed position in a lower collecting plate and then exits highly ionized through the front holes in continuous laminar flow. As a discharge current, an input voltage of AC 220 V, 50 Hz and the output high voltage −4.5 ± 0.5 KV were used, with the working current being <3 mA, at room temperature. The expected emission spectrum between 200 and 400 nm in the ultraviolet range wherein the spectral lines of NO (nitric oxide) in the range from 200 to 275 nm, OH^−^ (hydroxide ion) in the range from 275 to 310 nm, N_2_ (molecular nitrogen) in the range from 310 to 380 nm, and neutral and ionized atomic oxygen in the range from 380 to 400 nm.

The long-lived RONS, as NAIs emitted in the air media by aCAP-NR, were applied to enrich only the atmosphere for the experimental group mice cage. Measurements of atmospheric ion dilutions (ions/10^3^ cm^3^) within the cage of mice in the experimental group ranged from 1.5 in the peripheral cage area to 150 in the areas closer to aCAP source, suggesting the existence of different areas, reflecting that areas closer to the cage walls had the lowest level of ion dilutions. These atmospheric ion conditions ensure that outside the cage of the experimental group of mice, there was no NAI enrichment of breathable air at all; therefore, mice controls were not exposed to long-lived RONS enrichment. The aCAP-NR system was continuously applied in semi-enclosed environments with a temperature range of 10–40 °C and relative humidity of 35–75% (non-condensing). The applied level of ions was 150,000 ions/cm^3^, for uninterrupted exposure 24 h a day, throughout the whole experiment. The system’s technical specifications applied to the study included a supply voltage (230 V), with a frequency of 50 Hz and maximum power (525 W), under protection type Class 1 (EN 60601-1) [42] and IP21 protection rating.

### 2.3. Sample Preparation and Metabolomic Analysis

Metabolomics analyses were performed as previously described [23]. Briefly, 25 µL of whole blood or 2–5 mg of freeze-dried liver was mixed with the following amounts of internal standards dissolved in water (for a total of 500 µL water and 500 µL methanol). Details of the internal standards used and their concentrations are shown in Table 1. After the addition of 1 mL of chloroform, samples were homogenized using a TissueLyser II (Qiagen, Hilden, Germany) for 5 min at a frequency of 30 times per second. Samples were then centrifuged for 10 min at 18,000× *g*. The top layer of each sample, containing the polar phase, was then transferred to clean tubes and dried using a vacuum evaporator at 60 °C. Dried samples were reconstituted in 100 µL of 3:2 (*v*/*v*) methanol–water. Metabolites were analysed using a Waters Acquity ultra-high performance liquid chromatography system coupled to a Bruker Impact II™ Ultra-High Resolution Qq-Time-Of-Flight mass spectrometer (Billerica, MA, USA). Chromatographic separation of the compounds was achieved using a SeQuant ZIC-cHILIC column (PEEK 100 × 2.1 mm, 3 µm particle size, (Merck, Darmstadt, Germany) at 30 °C. The LC method consisted of a gradient running at 0.25 mL/min from 100% mobile phase B (9:1 acetonitrile–water with 5 mM ammonium acetate pH 8.2) to 100% mobile phase A (1:9 acetonitrile–water with 5 mM ammonium acetate pH 6.8) in 28 min, followed by a re-equilibration step at 100% B of 5 min. MS data were acquired using negative and positive ionization in full scan mode over the range of *m*/*z* 50–1200. Data were analysed using Bruker TASQ software version 2.1.22.3. All reported metabolite intensities were normalized to dry tissue weight, as well as to internal standards with comparable retention times and response in the MS.

### 2.4. Statistical and Pathway Analysis

Orthogonal Partial Least Squares Discriminant Analysis (OPLS-DA) was conducted to provide a comprehensive overview of the complete dataset following mean centred scaling, with the objective of identifying distribution patterns and elucidating metabolic differences between the study groups. To identify the best performing model, the Ropls-pvs R workflow was used, prioritizing predictive power while minimizing overfitting. This method refines the metabolite set to a key subset that enhances group separation and holds potential biomarker significance. Variable selection was guided by two main metrics: Variable Importance in Projection (VIP) and scaled loadings, (p [corr]). The optimal model was identified by aiming for high Q^2^ values, minimal discrepancies between R^2^Y and Q^2^, and low permutation *p*-values.

An unpaired, two-tailed t-test was used for statistical comparisons between groups, with a *p*-value < 0.05 considered indicative of statistical significance. To assess the magnitude of metabolite accumulation changes, the logarithm of the fold change was calculated. The OPLS-DA, volcano plot analysis, and *t*-tests were performed using R software (version 4.3.2), Ropls-pvs R package [43], and the MetaboAnalystR 4.0 package [44].

For pathway analysis, Bonferroni-corrected *p*-values were calculated using the quantitative enrichment analysis (QEA) module of MetaboAnalystR 4.0 [44], which is based on the global test algorithm. Differential metabolites from each group were evaluated with reference to the Kyoto Encyclopedia of Genes and Genomes (KEGG) database [45] and the Small Molecule Pathway Database (SMPDB) [46] to identify the most relevant biological pathways. KEGG pathway maps for the significant metabolites were visualized using the Pathview R package (version 1.42.0).

## 3. Results

A significant increase in the concentration of bioavailable NAIs was observed during environmental enrichment. The system maintained a continuous laminar flow of long-lived reactive oxygen and nitrogen species, ensuring sustained exposure to biologically active anions throughout the experimental period. These conditions contributed to the establishment of a nanoparticle-free environment.

Building upon previous proteomics findings in this experimental model, which revealed early molecular signatures of adaptation to atmospheric anion enrichment [20], the present study aimed to characterize the metabolome fingerprint induced by such environmental exposure. A targeted analysis of selected metabolites (Table 1) was conducted to investigate metabolome changes associated with mitochondrial function and antioxidant responses. Given the dynamic nature of the metabolome and its ability to reflect real-time biochemical activity, this approach offers deeper insights into the molecular phenotype driven by negatively charged atmospheric ions.

To this end, adult mice (19 weeks old) were exposed to a direct flow of NAI-enriched air generated by the aCAP-NR device for either 18 days (short-term exposure, TS) or 28 days (long-term exposure, TL), and compared to non-exposed control animals. The following sections describe the metabolome findings and the associated metabolome pathways altered under each exposure condition.

### 3.1. Changes in Metabolome Landscape

**Figure 1:**
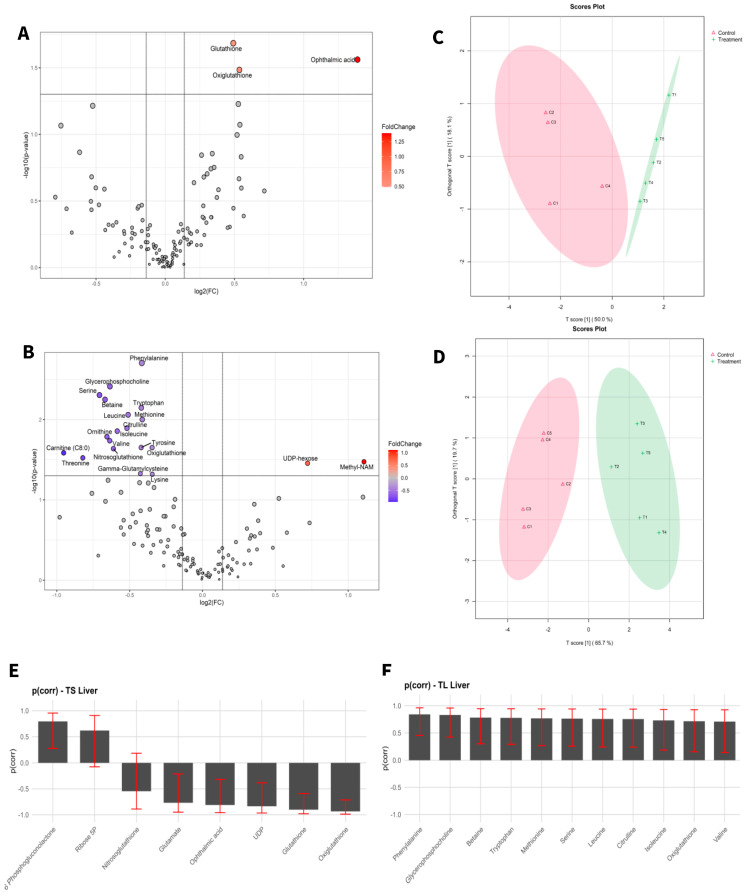
Statistical analysis of liver samples of short-term exposure vs. short-term control (**A**,**C**,**E**), and long-term exposure vs. long-term control (**B**,**D**,**F**). (**A**,**D**) Volcano plot with significant metabolites indicated. (**B**,**E**) OPLS-DA analysis. (**C**,**F**) Selected variables of the OPLS-DA model.

**Figure 2:**
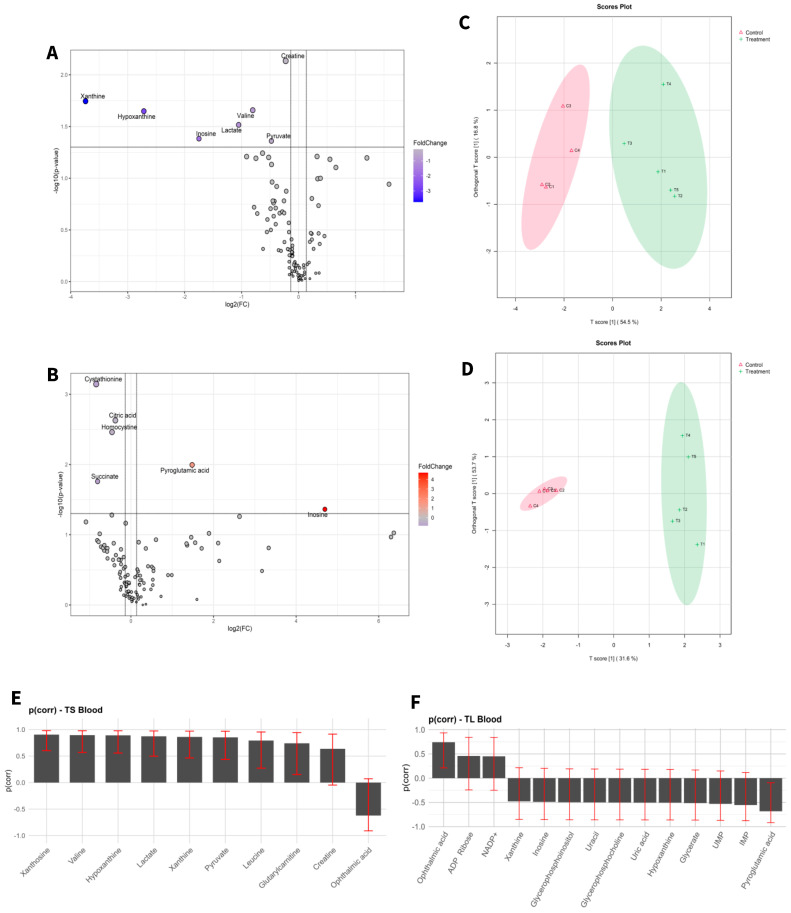
Statistical analysis of blood samples of short-term exposure vs. short-term control (**A**,**C**,**E**), and long-term exposure vs. long-term control (**B**,**D**,**F**). (**A**,**D**) Volcano plot with significant metabolites indicated. (**B**,**E**) OPLS-DA analysis. (**C**,**F**) Selected variables of the OPLS-DA model.

Target metabolic profiles were analysed from both liver tissue and blood of adult mice before and after exposure to aCAP-NR direct air flow free of nanoparticles and compared to non-exposed animals as controls. A total of 131 metabolites were identified in liver (Figure 1) and 111 in blood (Figure 2). The intensities of all detected metabolites are available in the Supplementary Tables S1 and S2 for liver and blood, respectively.

To visualize alterations in the metabolic profile, the results were presented as a volcano plot, which integrates statistical significance with fold change, highlighting metabolites showing a large magnitude of change (Figure 1A,B and Figure 2A,B).

As shown in Figure 1C,D and Figure 2C,D, the OPLS-DA models after variable selection demonstrated a pronounced separation between the control groups and those exposed to NAI exposome enrichment. These models were validated through permutation tests, yielding the following parameters in liver TS: R2Y: 0.731, Q2: 0.656; TL: R2Y: 0.983, Q2: 0.89, and in blood TS: R2Y: 0.798, Q2: 0.739; TL: R2Y: 0.981, Q2: 0.902. Figure 1E,F, as well as Figure 2E,F, illustrate the selected variables for the chosen models. Following variable selection through OPLS-DA models, a subsequent filtering step based on statistical significance was applied (*p* < 0.05). Table 2 and Table 3 enumerate the metabolites that met these criteria.

The results indicated that the highest number of significantly changed metabolites was observed in the liver of mice subjected to prolonged exposure compared to their controls. Conversely, short exposure induced the least pronounced changes in the liver of mice when compared to their controls.

### 3.2. Metabolome Pathway Analysis

**Figure 3:**
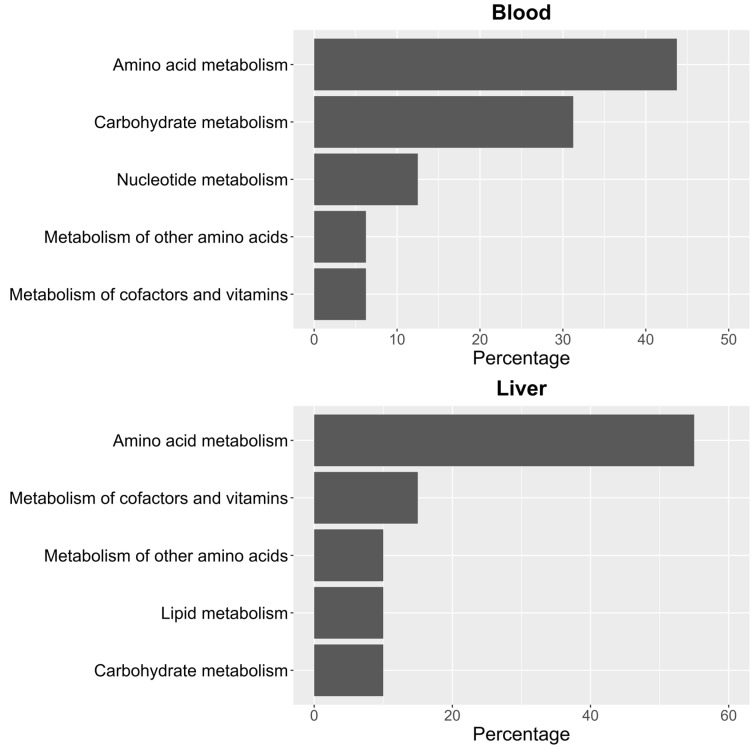
Percentage distribution of KEGG pathway groups for significant metabolites in blood and liver.

To identify significant KEGG and SMPDB pathways associated with the selected metabolites, quantitative enrichment analysis was performed using MetaboAnalystR 4.0 annotation tools. KEGG and SMPDB are curated databases that classify metabolic pathways based on genetic and biochemical knowledge. Pathway names used in this study reflect standardized annotations from these sources. The different metabolome patterns observed in blood and liver were categorized accordingly to highlight functionally relevant metabolic alterations. As shown in Figure 3, most of the identified pathways in both tissues belong to the amino acid metabolism category. In blood (Figure 3), this is closely followed by carbohydrate metabolism. In contrast, in liver (Figure 3), the next most impacted pathway is cofactor and vitamin metabolism, though its occurrence is significantly lower than that of amino acid metabolism.

It should be noted that pathway names reflect database annotations and may include reactions that are not necessarily active in mammals. In this context, pathway enrichment reflects statistically significant differences in the abundance of metabolites associated with a pathway, rather than full engagement of the entire biosynthetic or catabolic route.

**Figure 4:**
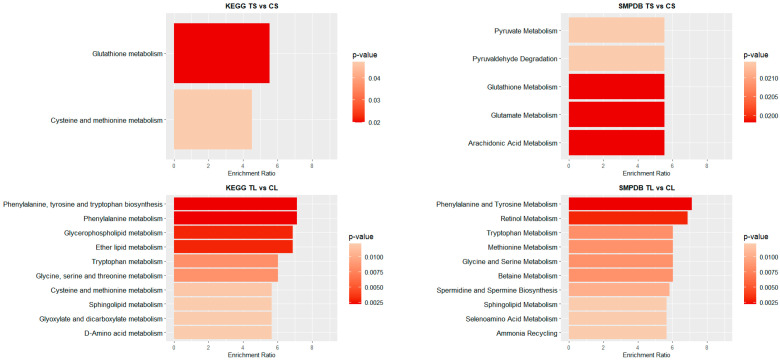
Pathway enrichment analysis of significant metabolites in liver samples. Bar plot illustrates the enrichment analysis of the top KEGG and SMPDB pathways with their *p*-values and enrichment ratio (number of results obtained in a metabolic pathway divided by the expected number of results). Deeper red colours represent pathways with the highest differential significance. TS: short-term exposure; CS: short-term control; TL: long-term exposure; CL: long-term control.

**Figure 5:**
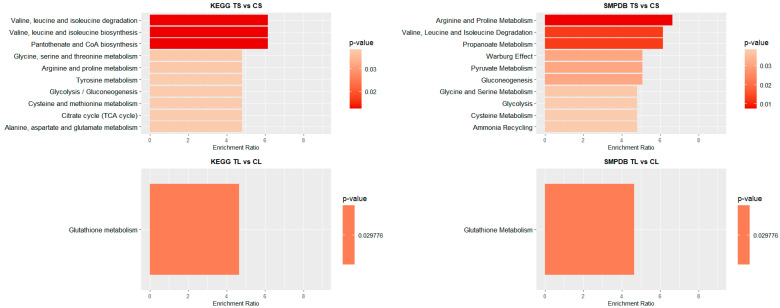
Pathway enrichment analysis of significant metabolites in blood samples. Bar plot showing the KEGG and SMPDB top pathways enrichment analysis, presenting their respective *p*-values and enrichment ratios (number of results obtained in a metabolic pathway divided by the expected number of results). Deeper red colours represent pathways with the highest differential significance. TS: short-term exposure; CS: short-term control; TL: long-term exposure; CL: long-term control.

Figure 4 and Figure 5 illustrate the top 10 pathways for each comparison. In liver (Figure 4), for shorter exposure times, analyses using both the KEGG and SMPDB reflect that glutathione metabolism is one of the most affected pathways. Additionally, notable alterations are observed in glutamate and arachidonic acid metabolism. For long-term exposure, the most significantly affected pathways include amino acid metabolism—such as phenylalanine, tyrosine, and tryptophan pathways—as well as vitamin A (retinol) metabolism.

**Figure 6:**
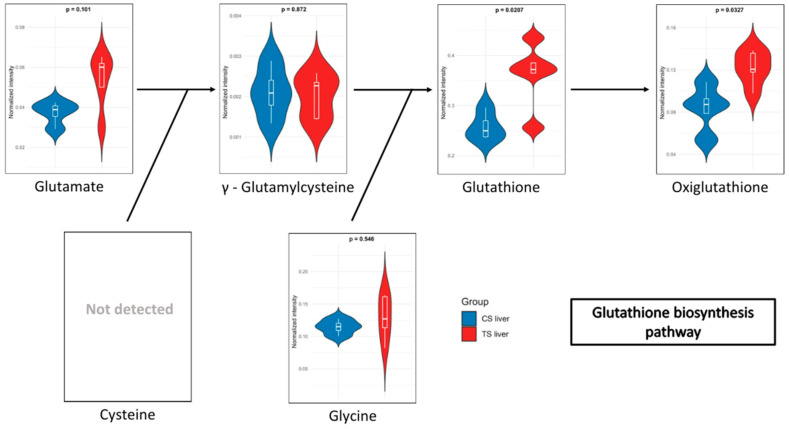
Relative abundance of metabolites involved in the glutathione biosynthesis pathway in liver after short-term NAI exposure. Data are shown as normalized intensity in control (CS, blue) and treated (TS, red) groups.

In the blood of long-term exposed mice (Figure 5), only a single pathway exhibits a statistically significant difference, glutathione metabolism, which was also observed in the liver of mice of the short-exposure group. Conversely, the short-exposure group shows a strong association of modified metabolites with the metabolism of several amino acids, including valine, leucine, isoleucine, arginine, and proline. Additionally, although with lower statistical significance, pathways related to carbohydrate metabolism—such as the TCA cycle and glycolysis/gluconeogenesis—are also affected. To further elucidate the metabolic alterations induced by NAI exposure, KEGG pathway maps for key metabolic pathways were generated to visualize the impact of exposure on blood and liver tissues. Significant metabolite alterations were identified for short-term exposure in blood, and for both short-term and long-term exposures in liver. The detailed pathway maps are presented in Supplementary Figures S1–S3: (1) glycolysis/gluconeogenesis in blood (short-term), (2) glutathione metabolism in liver (short-term), and (3) glutathione metabolism in liver (long-term). In addition, Figure 6 displays the KEGG-based representation of the glutathione biosynthesis pathway, highlighting the normalized abundance of key metabolites in liver tissue after short-term NAI exposure.

For the detailed results of the metabolome pathway analysis, please refer to the QEA file in the Supplementary Data (Tables S3–S6).

## 4. Discussion

In the present work, we investigate, for the first time, exposome interactions by analysing the association between the external exposome enriched by NAIs generated by the aCAP-NR system, and the endogenous metabolome, in C57BL/6 mature adult male mice, at different NAI exposure times and looking at the health outcomes by metabolome analysis. We have chosen the C57BL/6 strain since this is a genetically defined inbred mouse strain, and the most widely used in metabolic studies, as all individuals are healthy and genetically identical with minimal phenotypic variation, allowing for more statistically powerful analyses.

The exposome top-down strategy for analysing health status and finding causes of disease relies on samples of biospecimens to simultaneously investigate exposures originating both inside and outside the body [47]. This exposome study encompasses an exposure to exogenous NAIs and the analysis of the endogenous metabolites from blood and liver that are produced or altered in response to NAIs as external stressors. Although serum is often considered the gold standard for clinical studies, it has been suggested that blood should be preferred above serum for clinical metabolome studies as the serum metabolome may be substantially confounded by platelets [48]. In this study, all 19-week adult mice (equivalent to approximately 25 human years) were exposed to the same conditions of NAI enrichment as the main source of breathing air in comparison to controls. Monitoring the exposure included baseline analysis, without NAI enrichment, and after 18 and 28 days (equivalent to 3 and 4 human years, respectively) of NAI environmental enrichment using aCAP-NR. This was achieved through indirect CAP application, in which ionized air containing long-lived RONS (such as H_2_O_2_, NO_2_^−^, NO_3_^−^, and ONOO^−^) was delivered via a nanoparticle-free laminar flow, without direct contact with the plasma source. This configuration helps preserve the energy and reactivity of the anions, ensuring their bioavailability through inhalation or skin absorption. This approach allowed the study of the adaptative response capacity to a modified external exposome, especially when it is expected to find out cumulative biological effects produced by NAIs, as environmental enrichment influences reactivity by genetic and epigenetic mechanisms [49], induces a reduction in neurodegenerative markers [20], improves mitochondrial energy efficacy and antioxidant effects, and promotes changes in the respiratory transport chain in peripheral blood lymphocytes [4]. Initially, the understanding of NAI-induced adverse events was explored. The comparative analysis of treated and control mice at both exposure times (18 and 28 days) revealed no systemic or hepatic side effects, as well as no side effects in terms of behaviour, weight, exercise and explorative activity, fat accumulation disorder, dyslipidaemia, hyperglycaemia, and hyperinsulinemia, nor metabolic and hepatic damage. These exposomic adaptative responses observed in mice can be extrapolated to humans, revealing that continuous and prolonged NAI enrichment by aCAP-NR is a safe approach to also be used in humans, especially taking into account that, in humans, a continuous (24h/day) exposure to NAI enrichment is not possible. Nevertheless, although no adverse effects were observed in our study under either short- or long-term exposure conditions, further research is warranted to fully assess potential long-term effects and to investigate how NAIs may interact with other biological and environmental factors. Importantly, the distinct metabolome expression patterns identified in response to environmental enrichment open new avenues for precision medicine, enabling the discovery of novel biomarkers to detect early shifts in health status in response to external changes [50].

Exposome analysis incorporating biological responses requires multiple disciplines and tools to measure its high dynamism in time and space, the quantum nature of dose–response relationships, and the variability of individual responses with exposure history, age, exposure time, and co-exposures [51]. Therefore, incorporation of omics methods, such as the analysis of the metabolome, helps to characterize the molecular changes associated with the expected cumulative biological effects. Indeed, in this study, monitoring NAI exposure with biological responses for defining the functional exposome provides a better understanding of the relationship between NAIs (exogenous exposome) and metabolome outcomes (endogenous exposome), detected before and after NAI exposure. This is the first study that uses a metabolome targeted follow-up approach to evaluate the effect of exposome enrichment by NAIs using the aCAP-NR strategy. The current work focuses on the role of blood and liver metabolites from the endogenous metabolome by targeted metabolomics-based tools, knowing that the NAIs of the environment generated by aCAP-NR must be the unique driver of the individual’s metabolomic phenotypes in response to different exposure time strategies to untangle the interactions between similar external NAI exposures and an individual’s endogenous metabolites in comparison to non-exposure controls, and using statistical and computational methods associating the exposome to metabolome outcomes to achieve precision metabolomics, looking for markers that can be clinically actionable.

Using a metabolomics-based approach, we perform targeted tracking of a panel of blood and liver metabolites and compare metabolome data before and after aCAP-NR exposure. This allows us to understand changes in the healthy aging process through changes in metabolite levels due to the functional differences between different organs. In addition, the levels of metabolites in the same organ will be different during different time exposure strategies due to the different needs and responses of the body’s life activities [52].

In this study, KEGG pathway maps, as a reference curated database resource, allowed data interpretation at high-level functions in molecular interaction and reaction networks derived from the NAI external exposome interaction with the endogenous metabolome. Overall, KEGG analysis highlights the influence of NAI enrichment on metabolic pathways related to amino acids, carbohydrates, nucleotides, lipids, and cofactors and vitamins, both in blood and liver. The results of the targeted metabolome analysis in blood and liver of adult mice indicate that NAI enrichment by aCAP-NR significantly influences the endogenous exposome of the animals, leading to remarkable shifts in their metabolic profile. Metabolic pathway analysis in these two tissues also suggests that NAI enrichment by aCAP-NR can reach multiple tissues and have a significant impact across diverse metabolic pathways. These pathways are named according to KEGG and SMPDB annotations, and their inclusion reflects statistically enriched associations with detected metabolites. As such, they do not necessarily imply full functional activation of the corresponding biological routes in mammals.

Adult mice after short-term exposure to NAIs exhibited a reduction in lactate and pyruvate levels in blood when compared to controls. These two metabolites are key in boosting cellular energy and wound healing processes [53]. During periods of intense exercise, oxygen availability becomes a limiting factor, and the energy demands required to sustain muscle contraction frequently exceed the mitochondrial capacity to produce ATP through oxidative phosphorylation [54,55]. Under anaerobic conditions, in order to sustain glycolysis, pyruvate enters the alternative lactic fermentation pathway, wherein the cytosolic enzyme lactate dehydrogenase (LDH) converts pyruvate into lactate. The lactate produced in muscle tissue is then transported into the bloodstream, circulated, and subsequently absorbed by the liver. In the liver, LDH reconverts lactate into pyruvate, thereby supporting the citric acid cycle and gluconeogenesis. This entire process is known as the Cori cycle [56,57]. The observed decreases in blood lactate and pyruvate levels may reflect a more efficient utilization of energy, thus reducing the need for lactate production and, by extension, pyruvate production [57] and reducing the risk of lactic acidosis [58]. However, as both metabolites are tightly regulated and subject to rapid physiological fluctuations, their interpretation should be made cautiously and within the broader context of systemic metabolism.

In addition, a mild decrease in blood creatine levels is observed. Creatine plays a vital role in the rapid regeneration of ATP, which is essential for muscle contraction during intense physical activity. When muscles undergo adequate recovery following exercise, the need to break down creatine for energy diminishes. This may result in a modest reduction in blood creatine levels as the body optimizes its utilization and storage [58,59]. In this context, the reduction in valine levels is noteworthy, since valine, a branched-chain amino acid, plays a crucial role in muscle metabolism and energy production [60,61]. Such a decrease may suggest increased utilization by tissues, particularly in muscle, during exercise or recovery phases. This could signify effective post-exercise recovery, as the body becomes more efficient in using amino acids to fulfil energy and protein synthesis requirements, thereby facilitating enhanced muscle repair and growth [62,63]. Additionally, a reduction in purine metabolism was observed which can contribute to reactive oxygen species (ROS) generation via the activity of xanthine oxidase, which converts hypoxanthine into xanthine and subsequently into uric acid [64,65,66,67]. Therefore, the observed reduction in purine metabolism could also imply a decrease in oxidative stress. Furthermore, it has been reported that blood levels of hypoxanthine and xanthine are important indicators of metabolic efficiency. A reduction in the accumulation of these metabolites may thus point towards enhanced ATP production and a more efficient utilization of energy substrates [54,55]. These findings are consistent with previously published results, demonstrating an improvement in ATP production efficiency both in human peripheral blood lymphocytes (PBMCs) and mouse brain [4,20].

Regarding the metabolites detected in the liver, after short-term NAI exposure, an increase in glutathione (GSH) metabolism was observed. GSH is a key antioxidant that plays an essential role in maintaining cellular health and preventing chronic diseases. Low levels of GSH are associated with various inflammatory conditions, including metabolic syndrome, cardiovascular diseases, and neurodegenerative disorders [68,69,70]. As a potent antioxidant, GSH protects against oxidative stress, regulates cellular processes, and supports immune function [71]. In addition, oxidized glutathione (GSSG), commonly referred to as oxiglutathione, serves a critical function in the cellular antioxidant defence system. Reduced glutathione (GSH) and oxiglutathione collaboratively engage in a redox cycle that effectively neutralizes reactive oxygen species (ROS) and other free radicals, thereby protecting cells from damage [72]. Moreover, GSH depletion has been shown to induce ferroptosis and autophagy [73]. Ferroptosis is considered one of the most ancient and prevalent forms of regulated non-apoptotic cell death, characterized by iron-dependent lipid peroxidation and ROS derived from lipid sources. This process has been involved in both the pathology of degenerative diseases and tumour suppression [74]. Excessive lipid peroxidation is regarded as a critical feature of ferroptosis, which can be mitigated by the action of glutathione. In this context, NAIs generated by aCAP-NR could have a powerful antioxidant effect in liver lipoperoxidation, preventing ferroptosis-induced cell death, and maintaining a healthier liver function. Indeed, an increase in the levels of ophthalmic acid was also observed after short-term NAI exposure. This compound is associated with amino acid metabolism and is synthesized from cysteine and homocysteine. Elevated levels of ophthalmic acid may indicate that the liver is actively engaged in detoxifying harmful compounds, potentially as a response to oxidative stress or environmental contaminants [75,76]. Additionally, ophthalmic acid may influence the availability of cysteine, a crucial amino acid for glutathione synthesis. The observed increased levels of ophthalmic acid may thus correlate with enhanced glutathione production also found after short-term NAI exposure, as it provides the necessary precursors for its synthesis as previously described [71,76]. Both ophthalmic acid and glutathione are critical components of the antioxidant defence system. While glutathione protects cells from oxidative damage, ophthalmic acid may help regulate glutathione levels and contribute to the detoxification of ROS.

All these findings from short-term NAI enrichment when extrapolated to the equivalent exposure times in humans reveal that the exposure to NAIs by aCAP-NR for prolonged periods (up to 3 years) may improve the adaptative mitohormetic response in metabolic efficiency, leading to a more effective utilization of energy, muscle recovery, antioxidant resilience and liver detoxifying functions, and reinforce the healthier and preventive effects associated with NAIs-aCAP-NR exposure [4,20].

The second part of this exposome monitoring study was to examine which underlying metabolome factors are involved in the exposome interactions to long-term exposure to NAIs, in adult mice. In this sense, NAI enrichment reveals significant blood metabolic changes compared to their controls, especially in regard to two metabolites, pyroglutamic acid (pGlu) and inosine.

After long-term exposure to NAI enrichment, the blood levels of pyroglutamic acid showed a slight increase in adult mice. The metabolite pGlu, derived from glutamate, plays a role in amino acid metabolism and cellular redox balance, functioning as an antioxidant with a vital role in brain function and ammonia detoxification. On the other hand, highly elevated levels of pyroglutamic acid may be linked to increased oxidative stress, as it can accumulate as a byproduct of glutathione degradation [77,78]. Furthermore, the role of this compound in modulating inflammatory processes has also been documented [79]. Therefore, the observed slight increase in pyroglutamic acid levels may reflect an adaptive metabolic response to NAI exposure, potentially contributing to the regulation of redox balance and inflammatory processes.

Findings after long-term NAI enrichment with aCAP-NR reveal a significant elevation in blood levels of the nucleoside inosine with an important role in purine biosynthesis, gene translation, modulation of RNAs, and as a secondary metabolite in purine metabolism acting as a molecular messenger in cell signalling pathways [80]. Inosine may result from ATP degradation during intensive energetic processes, such as physical exercise or metabolic stress. Consequently, inosine can function as an antioxidant, mitigating oxidative stress [81,82]. Furthermore, it may act as a modulator of the inflammatory response elicited by cytokine release under stress conditions, thereby assisting in the regulation of immune function and inflammation [83,84]. Therefore, the increased inosine blood levels after NAI enrichment by aCAP-NR may represent an adaptive antioxidant resilience mechanism.

Liver metabolome changes in various amino acids are additionally recorded after long-term NAI enrichment, with most of them exhibiting very mild changes (fold change of less than one). Under physiological conditions, the liver serves a vital role in the synthesis of plasma proteins and the production of enzymes and other essential metabolic compounds [85,86,87]. Consequently, a slight decrease in the circulating levels of these amino acids may be attributed to their utilization in protein synthesis. Furthermore, a decrease in oxidized glutathione is also evident, without any direct impact on glutathione levels. As previously mentioned, GSSG is the oxidized form of glutathione, formed when GSH acts as an antioxidant and becomes oxidized to GSSG. The reduction in oxidized glutathione after NAI enrichment may indicate that the antioxidant system is more effective, and the cells are recovering a more balanced redox state following oxidative stress. Moreover, the GSH/GSSG ratio is an important indicator of cellular health, with lower ratios indicating oxidative stress conditions [71,88]. Overall, the accumulated evidence suggests that mature adult mice subjected to prolonged exposure to NAI enrichment may be pre-conditioning hormetic responses to effectively managed oxidative stress.

Consequently, after long-term NAIs-aCAP-NR exposure, the observed elevation in inosine levels as a byproduct of ATP degradation may be attributed to an enhanced energy consumption, necessary for the maintenance of homeostasis [89,90]. Moreover, both inosine and pyruvate may contribute to the regulation of inflammation and the immune response elicited by stress. The observed reduction in GSSG indicates that the organism is efficiently managing oxidative stress by mitigating the burden of ROS through the production of GSH. Ultimately, the liver may mobilize amino acids from reserves to satisfy metabolic demands, such as the synthesis of proteins required for cellular repair and energy production [91,92,93]. In summary, the metabolome changes after NAI enrichment may reflect an efficient adaptive mechanism for pre-conditioning metabolic response to oxidative stress, supporting hormesis and preserving cellular functions.

## 5. Conclusions

The exposome NAI enrichment induces a shift in the metabolome profile of blood and liver, suggesting a multisystemic impact. Specifically, short-term NAI enrichment by aCAP-NR appears to enhance both metabolic efficiency and resilience against oxidative stress. Long-term NAI exposure may be associated with the adaptive mechanism for pre-conditioning metabolic response to oxidative stress. The adaptive response observed after prolonged exposures suggests that initial exposure to NAIs may have induced an adaptation, potentially activating signalling pathways that stimulate antioxidant production and enhance future stress resilience. This adaptive process not only mitigates cellular damage but may also strengthen the organism’s overall resistance to stress, facilitating improved inflammatory regulation and more efficient energy resource management.

When extrapolating this NAI exposure monitoring data to the equivalent exposure times in humans, it may provide reasonable clues to suggest that the exposure to NAI enrichment by aCAP-NR is a long-term approach to facilitate a remote pre-conditioning hormetic response for efficient metabolic energy production, antioxidant resilience, and a healthier hepatic detoxification state, without unexpected adverse effects, making this approach a safe active preventive action against cumulative metabolome manifestations associated with oxidative stress during aging.

Nevertheless, further studies are warranted to identify the activated transcriptome pathways to determine the appropriate causal relationships in the hormesis processes driving the metabolome findings in this mature adult mice model exposed to NAI enrichment.

## Figures and Tables

**Table 1:** Internal standards used for metabolome analysis and their respective concentrations.

Internal Standards Compound	nmol	Internal Standards Compound	nmol
Adenosine-15N5-monophosphate	5.0	D5-glutathione	1.0
Adenosine-15N5-triphosphate	5.0	13C6-isoleucine	0.5
D4-alanine	0.5	D3-lactic acid	1.0
D7-arginine	0.5	D3-leucine	0.5
D3-aspartic acid	0.5	D4-lysine	0.5
D3-carnitine	0.5	D3-methionine	0.5
D4-citric acid	0.5	D6-ornithine	0.5
13C1-citrulline	0.5	D5-phenylalanine	0.5
13C6-fructose-1,6-diphosphate	1.0	D7-proline	0.5
Guanosine-15N5-monophosphate	5.0	13C3-pyruvate	0.5
Guanosine-15N5-triphosphate	5.0	D3-serine	0.5
13C6-glucose	10.0	D6-succinic acid	0.5
13C6-glucose-6-phosphate	1.0	D5-tryptophan	0.5
D3-glutamic acid	0.5	D4-tyrosine	0.5
D5-glutamine	0.5	D8-valine	0.5

**Table 2:** Mean ± standard deviation (SD) of metabolites significantly altered in liver samples. Only metabolites with *p* < 0.05 are shown. TS: short-term exposure; CS: short-term control; TL: long-term exposure; CL: long-term control.

**TS vs. CS**				
**Metabolites**	**TS (Mean ± SD)**	**CS (Mean ± SD)**	***p* Value**	**Log Fold Change**
Glutathione	13.195 ± 1.704	11.78 ± 2.115	0.02065847	0.49196
Ophthalmic acid	0.079 ± 0.038	0.035 ± 0.009	0.02743137	1.3896
Oxiglutathione	4.451 ± 0.266	3.709 ± 0.24	0.03274709	0.53576
**TL vs. CL**				
**Metabolites**	**TL (Mean ± SD)**	**CL (Mean ± SD)**	***p* Value**	**Log Fold Change**
Phenylalanine	28.285 ± 7.546	34.82 ± 9.783	0.00198513	−0.41637
Glycerophosphocholine	0.716 ± 0.11	0.901 ± 0.213	0.00386516	−0.63543
Serine	88.144 ± 25.328	112.205 ± 47.507	0.00494991	−0.70711
Betaine	0.117 ± 0.021	0.123 ± 0.025	0.00563138	−0.6689
Tryptophan	0.76 ± 0.095	0.85 ± 0.159	0.00712724	−0.41976
Leucine	0.165 ± 0.033	0.172 ± 0.036	0.00872041	−0.51078
Methionine	0.133 ± 0.036	0.15 ± 0.041	0.0099779	−0.41337
Citrulline	0.375 ± 0.048	0.439 ± 0.073	0.01278198	−0.51846
Isoleucine	0.585 ± 0.09	0.707 ± 0.141	0.01392599	−0.58514
Valine	28.285 ± 7.546	34.82 ± 9.783	0.0183183	−0.63669
Oxiglutathione	0.716 ± 0.11	0.901 ± 0.213	0.02235191	−0.34569

**Table 3:** Mean ± standard deviation (SD) of metabolites significantly altered in blood samples. Only metabolites with *p* < 0.05 are shown. TS: short-term exposure; CS: short-term control; TL: long-term exposure; CL: long-term control.

**TS vs. CS**				
**Metabolites**	**TS (Mean ± SD)**	**CS (Mean ± SD)**	***p* Value**	**Log Fold Change**
Creatine	0.068 ± 0.004	0.079 ± 0.005	0.00733447	−0.22613
Xanthine	0.016 ± 0.005	0.208 ± 0.181	0.01799637	−3.7395
Valine	0.588 ± 0.151	1.027 ± 0.243	0.021987	−0.8043
Hypoxanthine	0.005 ± 0.001	0.033 ± 0.02	0.02248012	−2.7161
Lactate	4.899 ± 1.743	10.156 ± 3.972	0.03046626	−1.0516
Pyruvate	0.42 ± 0.052	0.584 ± 0.135	0.04361697	−0.47708
**TL vs. CL**				
**Metabolites**	**TL (Mean ± SD)**	**CL (Mean ± SD)**	***p* Value**	**Log Fold Change**
Pyroglutamic acid	0.641 ± 0.341	0.229 ± 0.073	0.0101355	1.4824
Inosine	0.032 ± 0.045	0.001 ± 0.001	0.043386	4.6955

## Data Availability

Data are contained within the article and Supplementary Material.

## Supplementary Materials

The following supporting information can be downloaded at: https://www.mdpi.com/article/10.3390/biomedicines13040949/s1, Figure S1: KEGG pathway map of glycolysis/gluconeogenesis showing significantly altered metabolites after short-term exposure to NAIs in blood samples. Yellow nodes indicate upregulated metabolites, while blue nodes represent downregulated metabolites. Figure S2: KEGG pathway map of glutathione metabolism showing significantly altered metabolites after short-term exposure to NAIs in liver samples. Yellow nodes indicate upregulated metabolites, while blue nodes represent downregulated metabolites. Figure S3: KEGG pathway map of glutathione metabolism showing significantly altered metabolites after long-term exposure to NAIs in liver samples. Yellow nodes indicate upregulated metabolites, while blue nodes represent downregulated metabolites. Table S1: Combined dataset of metabolite intensities from positive and negative measurements in liver samples, comparing exposure conditions against their respective controls. Data are normalized using internal standards and curated for missing values. Table S2: Combined dataset of metabolite intensities from positive and negative measurements in blood samples, comparing exposure conditions against their respective controls. Data are normalized using internal standards and curated for missing values. Table S3: Pathway enrichment analysis of metabolites in short-term exposure in liver samples from KEGG and SMPDB, described in terms of the number of detected metabolites (Hits) and statistical values, including the Raw *p* (unadjusted *p*-value) and FDR (False Discovery Rate). Table S4: Pathway enrichment analysis of metabolites in long-term exposure in liver samples from KEGG and SMPDB, described in terms of the number of detected metabolites (Hits) and statistical values, including the Raw *p* (unadjusted *p*-value) and FDR (False Discovery Rate). Table S5: Pathway enrichment analysis of metabolites in short-term exposure in blood samples from KEGG and SMPDB, described in terms of the number of detected metabolites (Hits) and statistical values, including the Raw *p* (unadjusted *p*-value) and FDR (False Discovery Rate). Table S6: Pathway enrichment analysis of metabolites in long-term exposure in blood samples from KEGG and SMPDB, described in terms of the number of detected metabolites (Hits) and statistical values, including the Raw *p* (unadjusted *p*-value) and FDR (False Discovery Rate).
