# Supplementary material for: The Hormetic Adaptative Capacity and Resilience to Oxidative Stress Is Strengthened by Exposome Enrichment with Air Cold Atmospheric Plasma: A Metabolome Targeted Follow-Up Approach
Source: Biomedicines. 2025 Apr 12;13(4):949. doi: 10.3390/biomedicines13040949 (PMC12025095; doi:10.3390/biomedicines13040949)

Figure S1: KEGG pathway map of Glycolysis / Gluconeogenesis showing significantly altered metabolites after short-term exposure to NAIs in blood samples. Yellow nodes indicate upregulated metabolites, while blue nodes represent downregulated metabolites.

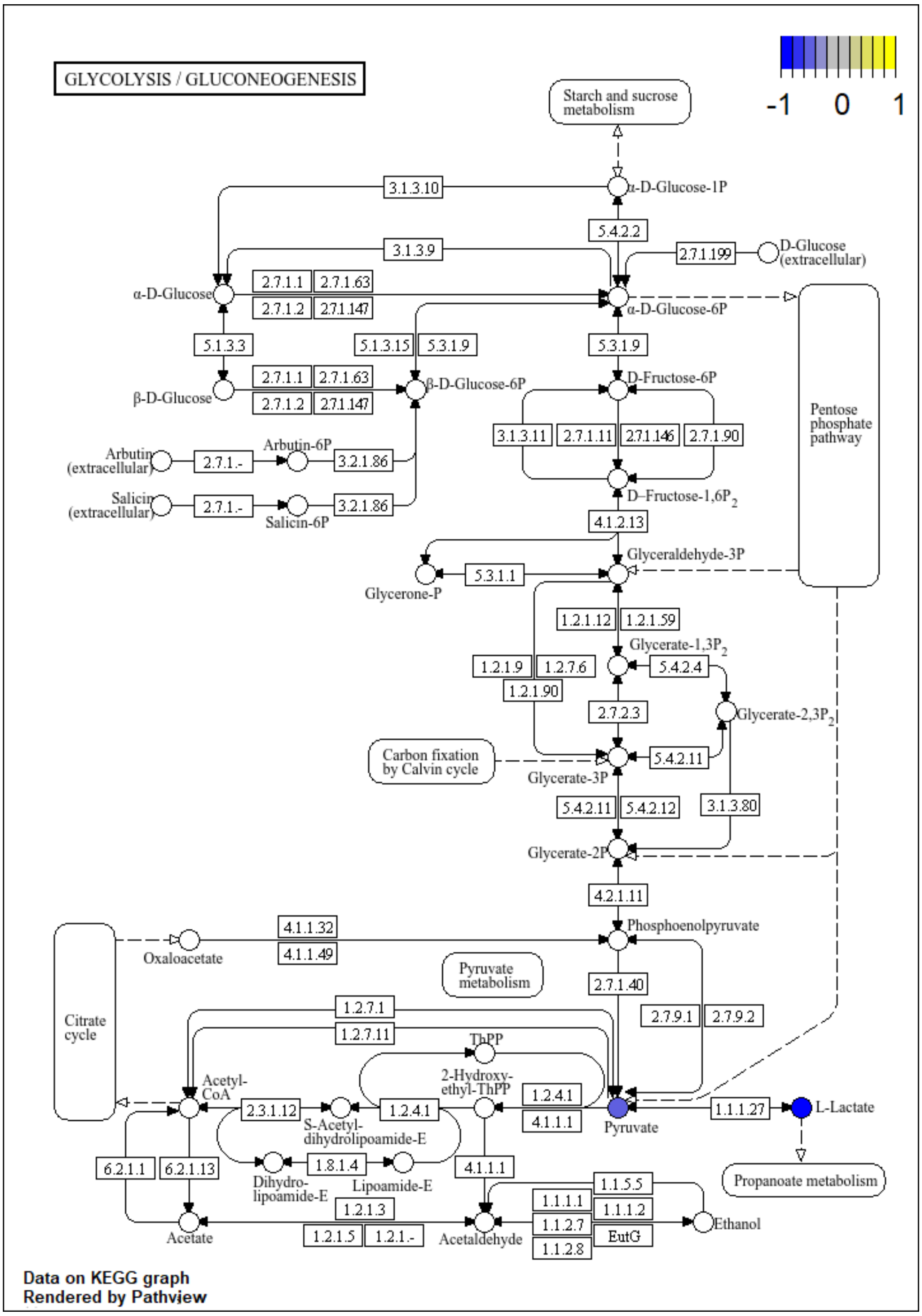

Figure S2: KEGG pathway map of Glutathione metabolism showing significantly altered metabolites after short-term exposure to NAIs in liver samples. Yellow nodes indicate upregulated metabolites, while blue nodes represent downregulated metabolites.

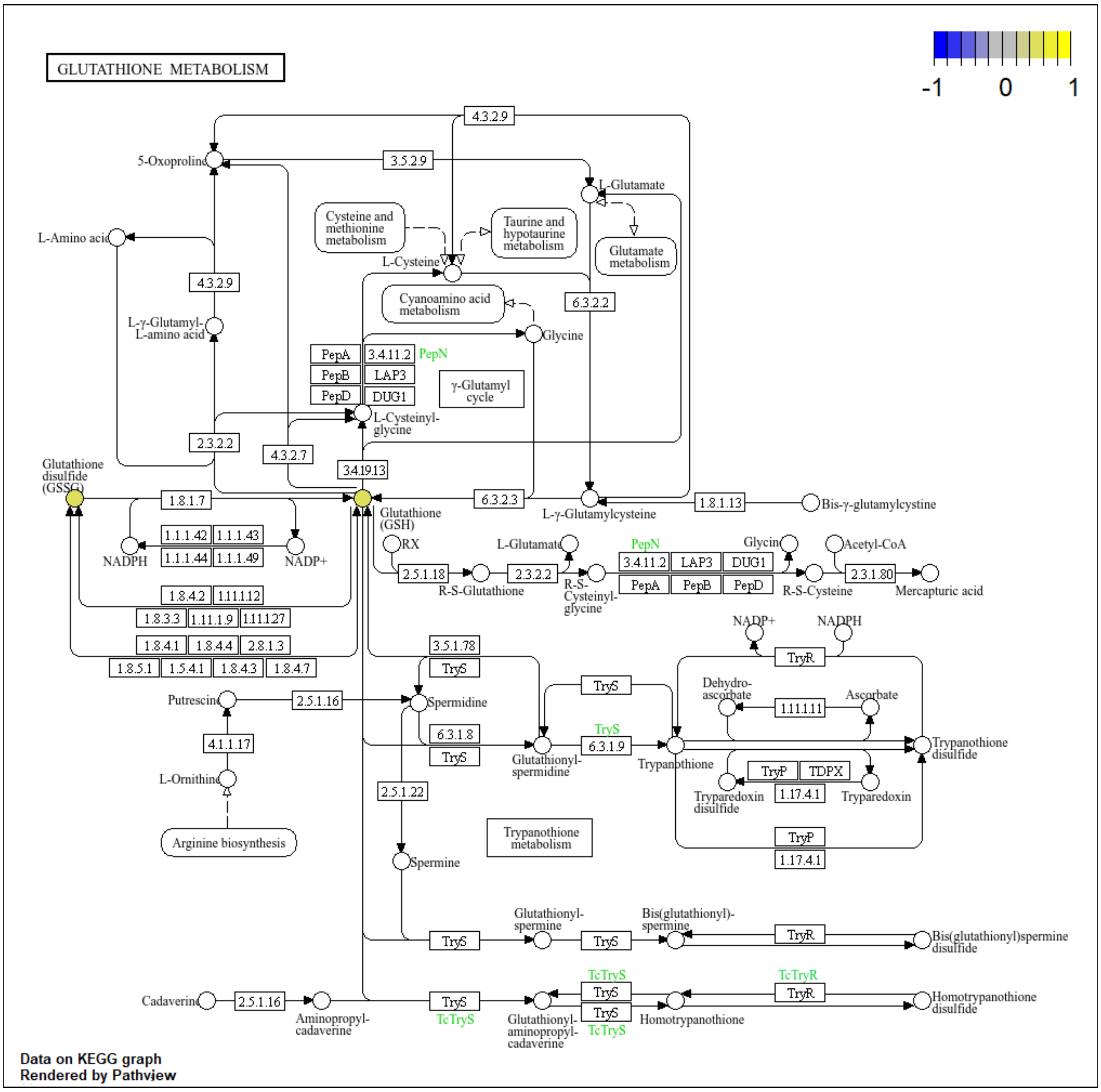

Figure S3: KEGG pathway map of Glutathione metabolism showing significantly altered metabolites after long-term exposure to NAIs in liver samples. Yellow nodes indicate upregulated metabolites, while blue nodes represent downregulated metabolites.

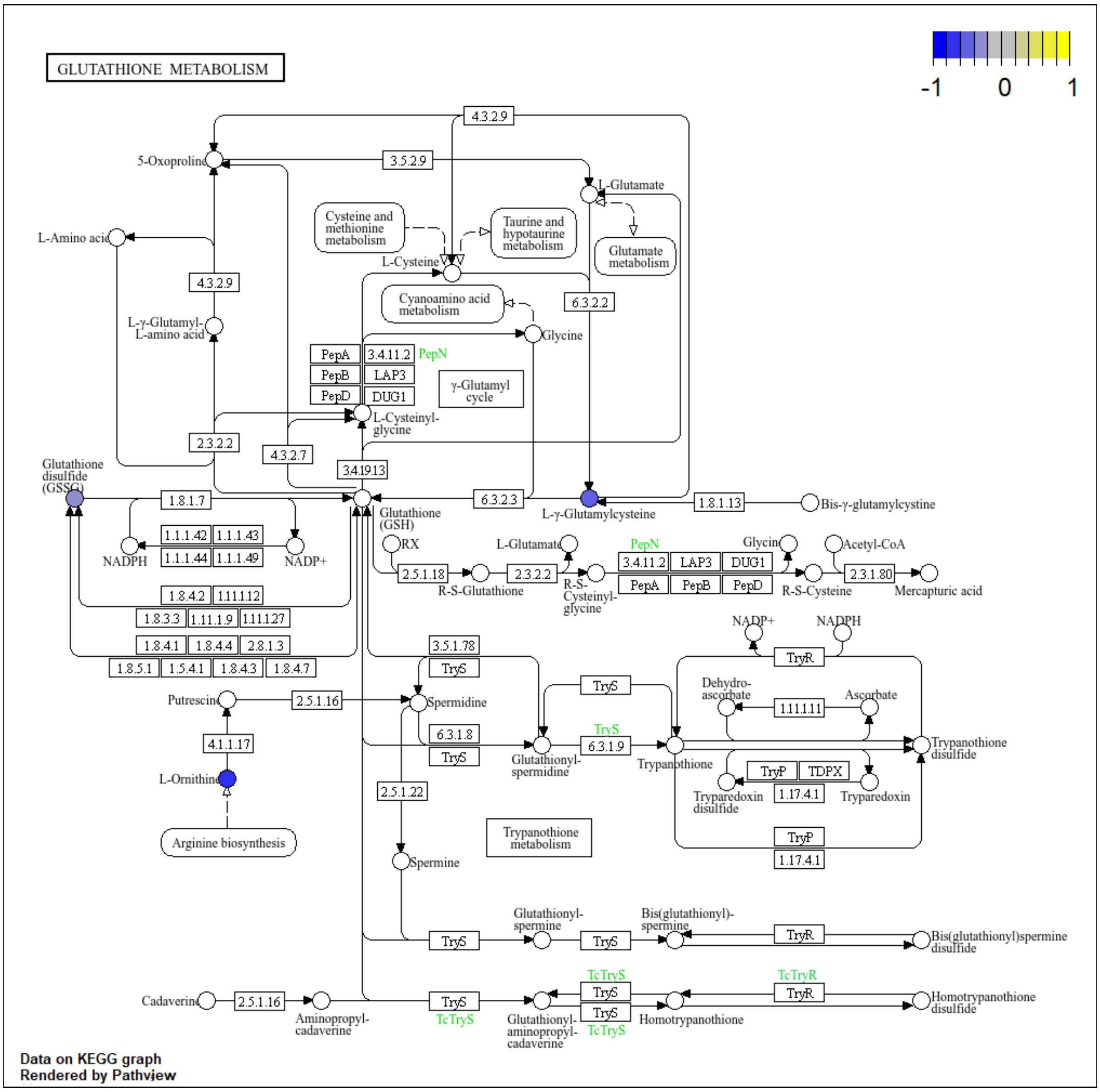

Supplement: Supplementary file 1 [file biomedicines-13-00949-s001.zip › biomedicines-3538117-supplementary-figures.pdf]
