# Supplementary material for: The Hormetic Adaptative Capacity and Resilience to Oxidative Stress Is Strengthened by Exposome Enrichment with Air Cold Atmospheric Plasma: A Metabolome Targeted Follow-Up Approach
Source: Biomedicines. 2025 Apr 12;13(4):949. doi: 10.3390/biomedicines13040949 (PMC12025095; doi:10.3390/biomedicines13040949)
Supplement: Supplementary file 1 [file biomedicines-13-00949-s001.zip › biomedicines-3538117-supplementary-tables.pdf]

Table S3: Pathway enrichment analysis of metabolites in short-term exposure in liver samples from KEGG and SMPDB databases, described in terms of the number of detected metabolites (Hits) and statistical values, including the Raw p (unadjusted p-value) and FDR (False Discovery Rate).

Pathway Name	Database	Total Compounds	Hits	Statistic Q	Raw p	FDR
Cysteine and methionine metabolism	KEGG	33	1	45.201	0.047264	0.047264
Glutathione metabolism	KEGG	28	2	55.356	0.019816	0.039631
Glutathione Metabolism	SMPDB	20	2	55.356	0.019816	0.021422
Glutamate Metabolism	SMPDB	48	2	55.356	0.019816	0.021422
Arachidonic Acid Metabolism	SMPDB	67	2	55.356	0.019816	0.021422
Pyruvate Metabolism	SMPDB	47	1	55.413	0.021422	0.021422
Pyruvaldehyde Degradation	SMPDB	10	1	55.413	0.021422	0.021422

Table S4: Pathway enrichment analysis of metabolites in long-term exposure in liver samples from KEGG and SMPDB databases, described in terms of the number of detected metabolites (Hits) and statistical values, including the Raw p (unadjusted p-value) and FDR (False Discovery Rate).

Pathway Name	Database	Total Compounds	Hits	Statistic Q	Raw p	FDR
Phenylalanine metabolism	KEGG	8	1	71.044	0.002196	0.011436
Phenylalanine, tyrosine and tryptophan biosynthesis	KEGG	4	1	71.044	0.002196	0.011436
Glycerophospholipid metabolism	KEGG	36	1	68.667	0.0030497	0.011436
Ether lipid metabolism	KEGG	20	1	68.667	0.0030497	0.011436
Tryptophan metabolism	KEGG	41	1	60.249	0.0082933	0.018195
Glycine, serine and threonine metabolism	KEGG	33	2	60.079	0.0084424	0.018195
Cysteine and methionine metabolism	KEGG	33	2	56.562	0.011826	0.018195
D-Amino acid metabolism	KEGG	15	1	56.538	0.01213	0.018195
Sphingolipid metabolism	KEGG	32	1	56.538	0.01213	0.018195
Glyoxylate and dicarboxylate metabolism	KEGG	31	1	56.538	0.01213	0.018195
Arginine biosynthesis	KEGG	14	1	55.16	0.013867	0.018909
Valine, leucine and isoleucine biosynthesis	KEGG	8	3	52.411	0.01677	0.01935
Valine, leucine and isoleucine degradation	KEGG	39	3	52.411	0.01677	0.01935
Glutathione metabolism	KEGG	28	1	50.194	0.02183	0.02339
Pantothenate and CoA biosynthesis	KEGG	20	1	48.981	0.024245	0.024245
Phenylalanine and Tyrosine Metabolism	SMPDB	27	1	71.044	0.002196	0.018489
Retinol Metabolism	SMPDB	35	1	68.667	0.0030497	0.018489
Tryptophan Metabolism	SMPDB	59	1	60.249	0.0082933	0.018489
Glycine and Serine Metabolism	SMPDB	59	3	60.079	0.0084424	0.018489
Methionine Metabolism	SMPDB	42	3	60.079	0.0084424	0.018489
Betaine Metabolism	SMPDB	21	2	60.079	0.0084451	0.018489
Spermidine and Spermine Biosynthesis	SMPDB	18	1	58.266	0.010201	0.018489
Ammonia Recycling	SMPDB	31	1	56.538	0.01213	0.018489
Selenoamino Acid Metabolism	SMPDB	27	1	56.538	0.01213	0.018489
Sphingolipid Metabolism	SMPDB	40	1	56.538	0.01213	0.018489

Pathway Name	Database	Total Compounds	Hits	Statistic Q	Raw p	FDR
Homocysteine Degradation	SMPDB	9	1	56.538	0.01213	0.018489
Phosphatidylethanolamine Biosynthesis	SMPDB	12	1	56.538	0.01213	0.018489
Arginine and Proline Metabolism	SMPDB	52	1	55.16	0.013867	0.018489
Urea Cycle	SMPDB	28	1	55.16	0.013867	0.018489
Aspartate Metabolism	SMPDB	35	1	55.16	0.013867	0.018489
Valine, Leucine and Isoleucine Degradation	SMPDB	59	3	52.411	0.01677	0.020963
Glutathione Metabolism	SMPDB	20	1	50.194	0.02183	0.022979
Glutamate Metabolism	SMPDB	48	1	50.194	0.02183	0.022979
Arachidonic Acid Metabolism	SMPDB	67	1	50.194	0.02183	0.022979
Propanoate Metabolism	SMPDB	42	1	48.981	0.024245	0.024245

Table S5: Pathway enrichment analysis of metabolites in short-term exposure in blood samples from KEGG and SMPDB databases, described in terms of the number of detected metabolites (Hits) and statistical values, including the Raw p (unadjusted p-value) and FDR (False Discovery Rate).

Pathway Name	Database	Total Compounds	Hits	Statistic Q	Raw p	FDR
Valine, leucine and isoleucine degradation	KEGG	39	1	61.409	0.012462	0.041479
Valine, leucine and isoleucine biosynthesis	KEGG	8	1	61.409	0.012462	0.041479
Pantothenate and CoA biosynthesis	KEGG	20	1	61.409	0.012462	0.041479
Glycine, serine and threonine metabolism	KEGG	33	2	48.077	0.037992	0.041479
Arginine and proline metabolism	KEGG	36	2	48.077	0.037992	0.041479
Glycolysis / Gluconeogenesis	KEGG	26	1	48.013	0.038517	0.041479
Citrate cycle (TCA cycle)	KEGG	20	1	48.013	0.038517	0.041479
Alanine, aspartate and glutamate metabolism	KEGG	28	1	48.013	0.038517	0.041479
Cysteine and methionine metabolism	KEGG	33	1	48.013	0.038517	0.041479
Tyrosine metabolism	KEGG	42	1	48.013	0.038517	0.041479
Pyruvate metabolism	KEGG	23	1	48.013	0.038517	0.041479
Glyoxylate and dicarboxylate metabolism	KEGG	31	1	48.013	0.038517	0.041479
Lipoic acid metabolism	KEGG	28	1	48.013	0.038517	0.041479
Purine metabolism	KEGG	70	2	45.818	0.044951	0.044951
Arginine and Proline Metabolism	SMPDB	52	1	66.436	0.0074334	0.040656
Propanoate Metabolism	SMPDB	42	1	61.409	0.012462	0.040656
Valine, Leucine and Isoleucine Degradation	SMPDB	59	1	61.409	0.012462	0.040656
Pyruvate Metabolism	SMPDB	47	2	50.785	0.031184	0.040656
Gluconeogenesis	SMPDB	33	2	50.785	0.031184	0.040656
Warburg Effect	SMPDB	57	2	50.785	0.031184	0.040656
Glycine and Serine Metabolism	SMPDB	59	2	48.077	0.037992	0.040656
Ammonia Recycling	SMPDB	31	1	48.013	0.038517	0.040656
Cysteine Metabolism	SMPDB	26	1	48.013	0.038517	0.040656
Glycolysis	SMPDB	23	1	48.013	0.038517	0.040656
Amino Sugar Metabolism	SMPDB	33	1	48.013	0.038517	0.040656

Pathway Name	Database	Total Compounds	Hits	Statistic Q	Raw p	FDR
Alanine Metabolism	SMPDB	17	1	48.013	0.038517	0.040656
Citric Acid Cycle	SMPDB	32	1	48.013	0.038517	0.040656
Urea Cycle	SMPDB	28	1	48.013	0.038517	0.040656
Glutamate Metabolism	SMPDB	48	1	48.013	0.038517	0.040656
Glucose-Alanine Cycle	SMPDB	13	1	48.013	0.038517	0.040656
Pyruvaldehyde Degradation	SMPDB	10	1	48.013	0.038517	0.040656
Transfer of Acetyl Groups into Mitochondria	SMPDB	22	1	48.013	0.038517	0.040656
Purine Metabolism	SMPDB	73	2	45.818	0.044951	0.044951

Table S6: Pathway enrichment analysis of metabolites in long-term exposure in blood samples from KEGG and SMPDB databases, described in terms of the number of detected metabolites (Hits) and statistical values, including the Raw p (unadjusted p-value) and FDR (False Discovery Rate).

Pathway Name	Database	Total Compounds	Hits	Statistic Q	Raw p	FDR
Glutathione metabolism	KEGG	28	1	46.533	0.029776	0.059551
Purine metabolism	KEGG	70	1	23.507	0.15553	0.15553
Glutathione Metabolism	SMPDB	20	1	46.533	0.029776	0.059551
Purine Metabolism	SMPDB	73	1	23.507	0.15553	0.15553
